# Bryophytes of the Serra dos Órgãos National Park: Endemism and Conservation in the Atlantic Forest

**DOI:** 10.3390/plants14152419

**Published:** 2025-08-04

**Authors:** Jéssica Soares de Lima, Allan Laid Alkimim Faria, Mateus Tomás Anselmo Gonçalves, Denilson Fernandes Peralta

**Affiliations:** 1 Instituto de Pesquisas Ambientais, Avenida Miguel Stéfano, 3687, São Paulo 04301-102, SP, Brazil; mateus.tomas2011@gmail.com (M.T.A.G.); denilsonfperalta@gmail.com (D.F.P.); 2 Instituto de Biologia, Universidade de Brasília, Campus Darcy Ribeiro, Asa Norte, Brasília 70910-900, DF, Brazil; allanlaid@gmail.com

**Keywords:** endangered species, highlands, liverworts, mosses

## Abstract

This study presents a comprehensive inventory of bryophytes in Serra dos Órgãos National Park (PARNASO), aiming to evaluate species richness, floristic composition and threatened taxa. Despite the state of Rio de Janeiro being one of the most extensively sampled regions for bryophytes in Brazil, detailed surveys of its conservation units remain scarce. Data were obtained through bibliographic review, herbarium specimen analysis, and new field collections. A total of 504 species were recorded, belonging to 202 genera and 76 families. The park harbors three locally endemic species, eight endemic to Rio de Janeiro, and sixty-nine species endemic to Brazil. Additionally, eleven species were identified as threatened, comprising seven Endangered (EN), two Critically Endangered (CR), and two Vulnerable (VU) according to the IUCN guidelines. PARNASO includes four distinct ecosystems along an altitudinal gradient: sub-montane forest (up to 500 m), montane forest (500–1500 m), upper-montane forest (1500–2000 m), and high-altitude fields (above 2000 m). Montane Forest showed the highest species richness, followed by high-altitude fields, upper-montane forest, and sub-montane forest. The findings highlight PARNASO’s importance in preserving bryophyte diversity in a highly diverse yet understudied region. This work contributes valuable baseline data to support conservation strategies and future ecological studies in Atlantic Forest remnants.

## 1. Introduction

Serra dos Órgãos National Park (PARNASO), established on November 30th, 1939, holds the distinction of being the third national park created in Brazil. This protected area is situated within the Atlantic Forest domain, specifically in the Serra do Mar massifs and called “Órgãos” in Portuguese because its peaks resemble and are named as “pipe organs”. The Serra do Mar is an extensive mountain range approximately 1500 km along the Brazilian coast, from the northern Santa Catarina State to Rio de Janeiro State [1,2].

PARNASO currently represents one of the most important remaining biodiversity hotspots in Rio de Janeiro State, since its ecosystems are one of the largest centers of flower plant endemism in the Atlantic Forest [3,4]. It also plays a vital role in preserving essential water sources that feed two main watersheds—Paraíba do Sul and several streams of the Baía de Guanabara [5,6,7,8].

The Atlantic Forest domain is one of the most species-rich forests in the tropics [4,9,10]. It is also recognized for having high levels of biodiversity and endemism, expressed via its complex structural environments [11,12]. Tropical forests harbor the highest diversity of bryophyte species in the world, not only due to their structural complexity and microhabitat heterogeneity, but also because of favorable climatic conditions such as high humidity, frequent precipitation, and relatively stable temperatures [13,14]. Some authors have reported that this diversity may vary along the altitudinal range, especially concerning structural and floristic composition [15,16,17,18].

The Atlantic Forest within PARNASO comprises well-preserved forests characterized by four distinct vegetation formations based on altitude, climate, and soil conditions, among other factors [6,19,20,21,22]. In the lower parts around Sede Guapimirim, sub-montane forest prevails, while near Sede at Teresópolis, the forest transitions from montane forest to upper-montane forest, culminating above 2000 m.a.s.l. and high-altitude fields along the trail from Teresópolis to Petrópolis [19].

The species list of the vascular plants in Flora Organensis [19] is strongly based on the work of 18th-century botanists and on collections made especially by A.C. Brade (1881–1971) and C.T. Rizzini (1921–1992) between the 1930s and 1950s of the 20th century. These collectors also recorded bryophytes, and the specimens were studied by Bartram (1954) [19], who identified 146 species. Most bryophyte specimens from Serra dos Órgãos are deposited in the Herbarium of Jardim Botânico do Rio de Janeiro (RB).

Bryophytes from PARNASO were not sufficiently explored, and even considering that, though several researchers collected and published new taxa, the study was dispersed as examined material in taxonomical revisionary work. For example, Faria et al. (2021) [23] attempted to re-collect species founded by Glaziou in Serra dos Órgaos and the taxonomic reclassification of an Atlantic Forest endemic, and Peralta et al. (2020) [24] reported species of the Andreaceae family from the region.

There is no other information on bryophytes in PARNASO; thus, there is no detailed data on the floristic structure and composition of bryophytes. A comprehensive species list will contribute to filling a significant knowledge gap for the state of Rio de Janeiro and support the assessment of threatened bryophyte species. In addition, the revision of herbarium specimens and the incorporation of new collections will help stimulate further research on the ecology and biogeography of bryophytes within this important national park. This work aims to provide an inventory of the bryophyte species in PARNASO and discuss the following topics: (i) species richness and floristic composition; (ii) substrate preferences; and (iii) endemism and conservation status.

## 2. Results

### 2.1. Species Richness and Floristic Composition

The present study documented 504 species in 202 genera and 76 families (Table 1). The Serra dos Órgaos National Park preserves 57% of the known bryophyte flora of Rio de Janeiro State and 31% of Brazil. A total of 3000 specimens were analyzed, resulting in the identification of 428 species and an additional 79 species (indicated by * in Table 1) that remain known only from the literature.

Three species are locally endemic, being known only from the type specimens collected over 100 years ago: *Southbya organensis* (Lützelburg 6003 p.p. [holotype JE]), *Plagiochila pseudoradicans* (Lützelburg 6029b [holotype JE]) and *Dicranodontium pulchroalare* ssp. *brasiliense* (Lützelburg 1519 [holotype JE]). Additional field research is urgently needed in order to obtain new records and data of these three endemic species.

The most species-rich families of liverworts are Lejeuneaceae (65 spp.) and Lepidoziaceae (16 spp.), while for moss they are Leucobryaceae (30 spp.) and Sphagnaceae (25 spp.). A total of 8 species of hornworts were found, distributed among the families Dendrocerotaceae (4 spp.), Anthocerotaceae (2 spp.) and Nothotyladaceae (2 spp.). The most species-rich genera were *Campylopus* (Leucobryaceae, 23 spp.); *Plagiochila* (Plagiochilaceae, 21 spp.); *Fissidens* (Fissidentaceae, 20 spp.); *Lejeunea* (Lejeuneaceae, 16 spp.); and *Metzgeria* (Metzgeriaceae, 14 spp.). Considering the samples analyzed, the most common species with the largest number of records were *Campylopus arctocarpus* (7 specimens); *Lejeunea acanthogona* (7); *Lejeunea flava* (7); *Radula nudicaulis* (7); *Bryum capillare* (6); *Fissidens wallisii* (6); *Lejeunea serpillifolioides* (6); *Rigodium toxarion* (6); *Trichocolea brevifissa* (6); and *Zelometeorium ambiguum* (6).

### 2.2. Substrate Preferences

**Figure 1:**
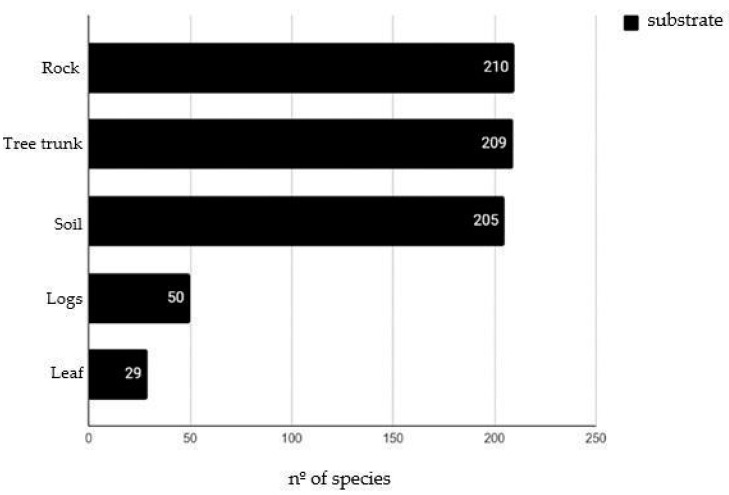
Substrates colonized by bryophytes in Serra dos Órgãos National Park.

The bryophyte species predominantly colonized rocks (210 spp.), followed by tree trunks (209 spp.), soil (205 spp.), logs (50 spp.), and leaves (29 spp.) (Figure 1).

The availability of trees within the forests of PARNASO clearly influences the bryophyte species occurrence. Mosses predominantly colonize tree trunks, with only four species being found on leaves: *Paraleucobryum longifolium* (Dicranaceae); *Philophyllum tenuifolium* (Hookeriaceae); *Toloxis imponderosa* (Meteoriaceae); and *Schlotheimia tecta* (Orthotrichaceae).

The lowest bryophyte colonization was for leaves (epiphyllous). The leaf-colonizing species most frequent were the leafy liverworts of the family Lejeuneaceae (21 spp.), followed by Radulaceae (2 spp.) and the thallose liverwort family Metzgeriaceae (2 spp.). According to Gradstein et al. (2001) [17] and Ilkiu-Borges & Lisboa (2004) [57], species of Lejeuneaceae can colonize different types of substrates, but their occurrence on leaves is indicative of preserved native areas.

### 2.3. Endemism and Conservation Status

The Serra dos Órgaos National Park protects three species locally endemic, eight from Rio de Janeiro state, and 69 Brazilian (Table 2). Endemism and species richness are high with an increase in altitude, as was also found in Itatiaia National Park, located in Serra da Mantiqueira [18].

Of the 28 species of Brazilian endemic bryophytes exclusive to high-altitude fields, 12 belong to the family Sphagnaceae (42%). The species *Sphagnum luetzelburgii* and *S. sehnemii* are endemic to Brazil, occurring in the states of Paraná, Santa Catarina, Rio Grande do Sul, and Rio de Janeiro. However, in Rio de Janeiro State, these species are restricted to mountains of the Atlantic Forest and have only been reported for PARNASO by Costa (2021) [58] and not by the present work.

The lower number of endemic species recorded in sub-montane forest probably reflects the effects of ecological and biogeographic processes, as well as human interference [18]. Among the 19 endemic species exclusively found in montane forest, 13 are liverworts and 6 are mosses. The predominant liverwort family is Lejeuneaceae, with nine species, followed by Metzgeriaceae with three and Jamesoniellaceae with two.

We have re-collected some species considered rare, including *Bryum limbatum*. Ochi (1980) [59] cites a collection from *Metzgeria furcata*, collected by Bartram (1954) [19]; *Sphagnum cuspidatum*, collected by Luetzelburg in 1923; and *Andreaea acutifolia*, collected by Mueller in 1845. *Andreaea acutifolia* is always mistakenly identified as *A. rupestris*, the two differing mainly by perichaetial leaves [24]. The species *S. cuspidatum* is widespread in Brazil [60], but has been collected only once in PARNASO.

**Figure 2:**
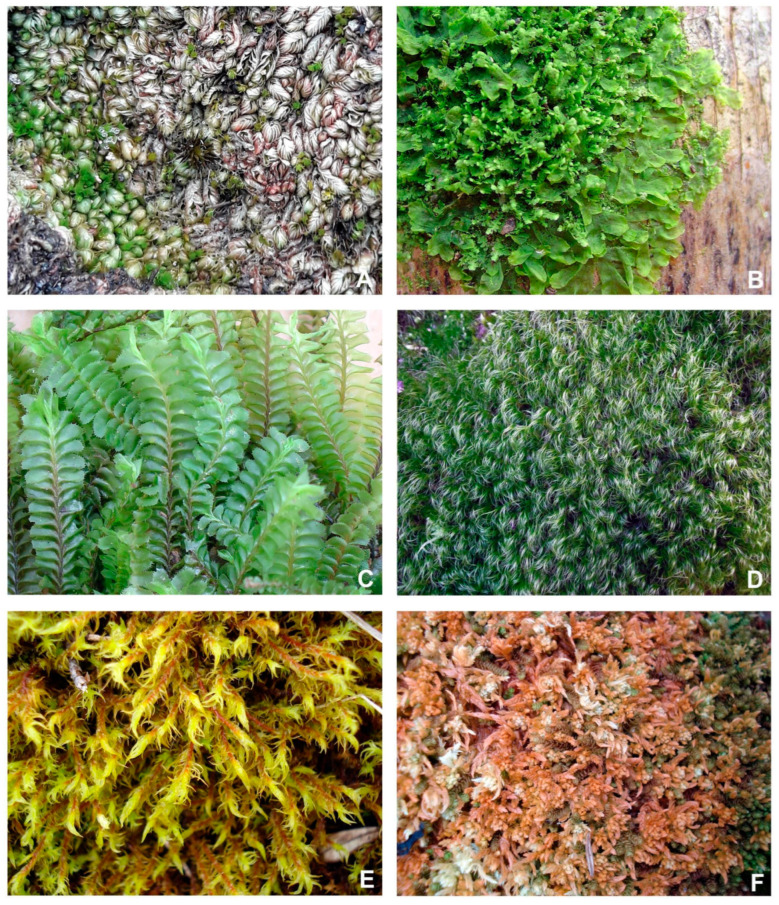
Pictures of some species indicated as threatened in Serra dos Orgaos National Park. (**A**) *Gongylanthus liebmannianus* (Lindenb. & Gottsche) Steph., (**B**) *Metzgeria hegewaldii* Kuwah., (**C**) *Plagiochila boryana* Gottsche ex Steph., (**D**) *Atractylocarpus longisetus* (Hook.) E.B.Bartram, (**E**) *Leptodontium wallisii* (Müll.Hal.) Kindb., (**F**) *Sphagnum perforatum* Warnst.

The Serra dos Orgaos Park also protects eleven of the species indicated as threatened by CNCFlora and Flora e Funga do Brasil (Figure 2 and Table 2) in the Rio de Janeiro state: seven as Endangered (EN), two as Critically Endangered (CR) and two as Vulnerable (VU). Among these threatened species, three are known only from the type material, and were not found in the field trips. *Southbya organensis* was described by Herzog (1949) [61] and is classified as Critically Endangered (CR) by CNCFlora (2012) [58] due to its distribution being restricted to Serra dos Órgãos, specifically Morro do Açu peak, which has excessive tourism and recurrent arson instead. *Plagiochila pseudoradicans* and *Dicranodontium pulchroalare* ssp. *brasiliense* were described by Herzog (1949) [61], and neither was assessed by CNCFlora (2012) [62] because they were considered as Data Deficient (DD) at that time.

## 3. Discussion

The Serra dos Órgãos National Park (PARNASO) exhibits remarkable bryophyte diversity. This richness highlights the park as a key conservation area for bryophytes in the Atlantic Forest. These findings suggest that even in areas already considered well-studied, significant knowledge gaps remain, highlighting the dynamic nature of bryophyte diversity and distribution.

One important factor contributing to this diversity is the presence of forested areas with abundant tree trunks, which serve as critical substrates for epiphytic bryophytes. The architectural, morphological, phenological, and chemical traits of different tree species create varied microhabitats that support a wide range of bryophyte taxa [57,63]. Shade-loving epiphytes, in particular, are commonly found in these forest environments but are highly sensitive to environmental disturbances. As such, they are often among the first species to disappear following canopy opening or habitat degradation [64], making them reliable indicators of forest health.

In contrast, mosses exhibit a broader ecological amplitude due to their desiccation tolerance and structurally complex adaptations, such as costa development, hyaline leaf apices, papillae, and specialized conducting tissues like hydroids and leptoids [29,65,66]. These features enable many mosses to colonize exposed and drier substrates, including rocky outcrops. Similar patterns of moss dominance on rock surfaces have been observed in other highland regions of Brazil, such as Chapada Diamantina [67,68], Serra do Caraça [69], and Serra da Canastra [70].

The high-altitude ecosystems of PARNASO further enhance bryophyte diversity by creating isolated and unique habitats. Mountain summits, often compared to ecological islands due to their environmental isolation, support specialized flora adapted to harsh conditions such as strong winds, low temperatures, and nutrient-poor soils [71,72,73]. These rupestrian fields form natural refugia, acting as barriers to typical forest species and favoring the emergence of narrow endemics and rare taxa [74,75,76].

Overall, the findings of this study underscore the ecological importance and conservation value of Serra dos Órgãos National Park. Alongside Itatiaia National Park, PARNASO remains one of the most critical remnants of Atlantic Forest in Rio de Janeiro and Brazil, particularly in terms of bryophyte conservation. The park’s diverse topography, wide range of microhabitats, and climatic heterogeneity—marked by high altitudes, low temperatures, and high light availability—create optimal conditions for bryophyte diversity. Furthermore, the presence of endemic and threatened species reinforces the park’s role as a conservation priority. Such studies are valuable as they reveal the significance of endemism and species richness, crucial factors in determining conservation priorities [77], and emphasize the importance of preserving Serra dos Órgãos National Park.

## 4. Materials and Methods

### 4.1. Study Area

**Figure 3:**
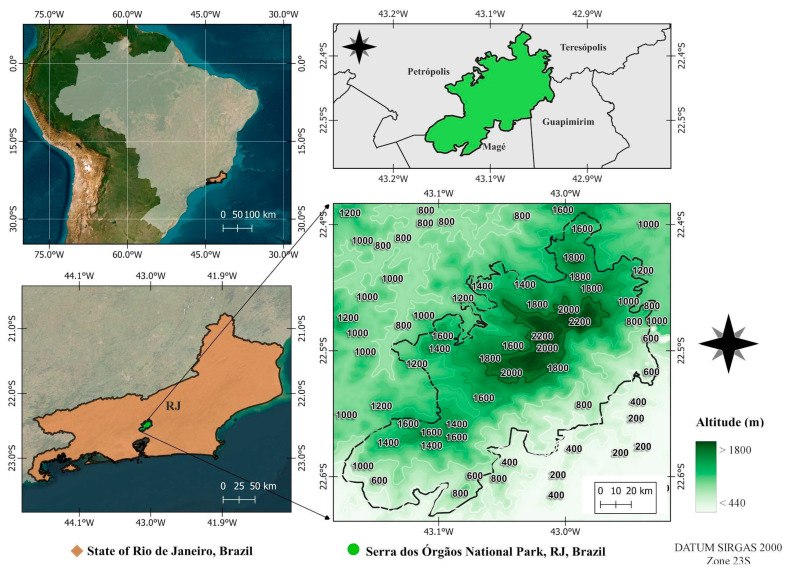
Location of the Serra dos Órgãos National Park, Rio de Janeiro State, Brazil.

Serra dos Órgãos National Park (PARNASO) is located in the highlands region of Rio de Janeiro State, Brazil, ranging the municipalities of Teresópolis, Petrópolis, Guapimirim and Magé (22°23′36.96″–22°34′57.72″ S and 43°10′57.72″–42°58′43.68″ W). It is a protected area with 20,024 hectares of Atlantic Forest in a central position in the Serra do Mar Ecological Corridor. Ranging in altitude from 80 to 2275 m.a.s.l. (Figure 3), it is the highest portion of the entire Serra do Mar [2]. The climate is tropical and super humid (80–90% relative humidity), with the annual average temperature ranging from 13 to 23 °C, although minimums can reach −5 °C during winter above 800 m.a.s.l. [6,8,74]. The average annual rainfall ranges from 1500 mm to 3000 mm, with higher concentrations of rain during summer (December to March) and a dry season during winter (June to August), while heat is well distributed throughout the year [78,79].

**Figure 4:**
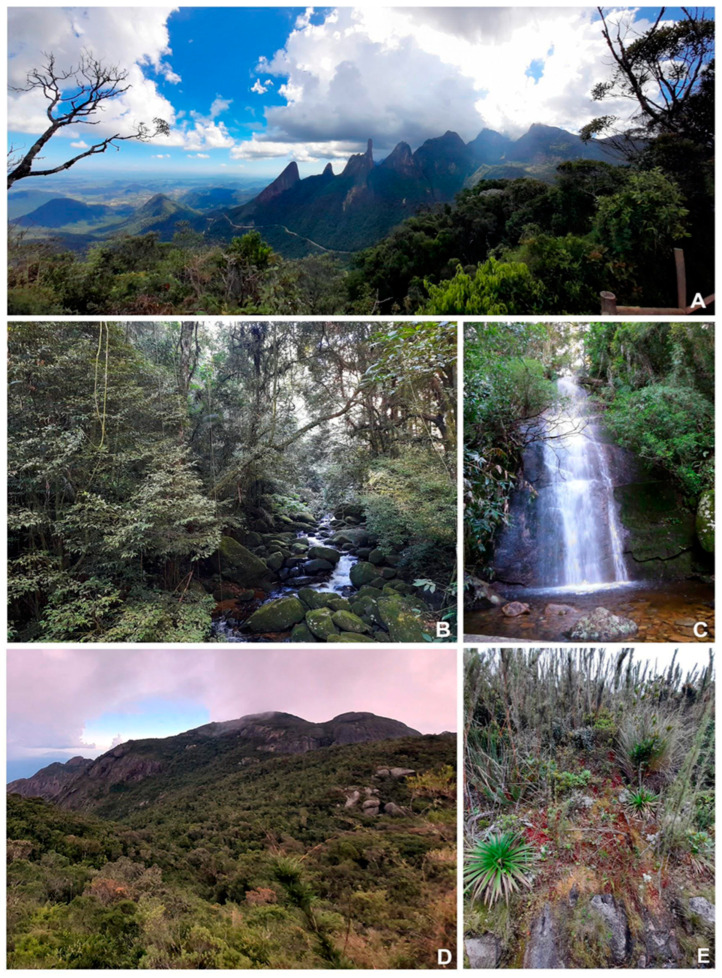
Collection sites in Serra dos Órgãos National Park. (**A**) Trail of the “Mirante”, (**B**) aspect of the montane forest with streams, (**C**) “Véu da Noiva” waterfall, (**D**) aspect of the upper-montane forest, (**E**) aspect of high-altitude fields, with *Sphagnum* among *Chusquea* sp.

The vegetation classification adopted follows Veloso et al. (1991) [20] and Safford (1999) [80]: Sub-montane Forest (below 600 m.a.s.l., typically evergreen, with a high canopy and rich floristic diversity; dominant tree species include *Protium* spp., *Virola* spp., and members of the Myrtaceae and Fabaceae families); montane forest (600–1500, characterized by shorter trees and more frequent presence of cloud cover; common tree species include *Clethra* spp. and various species of Melastomataceae); upper-montane forest (above 1500, vegetation here is more stunted, often with gnarled trees adapted to frequent mist and lower temperatures). Dominant elements include *Weinmannia paulliniifoli* and *Drimys brasiliensis*, and high-altitude fields (above 1800–2000) are open grassland formations interspersed with shrubs, herbs, and occasional low woody elements. Common species include *Vellozia* spp., *Paepalanthus* spp., and *Eryngium* spp. (Figure 4).

### 4.2. Studied Taxa

The species list developed here is based on (i) a bibliographic survey conducted by consulting published volumes of major Brazilian botanical journals, as well as international bibliographic databases (Web of Science, SciELO, and Google Scholar), in search of peer-reviewed articles; the search focused on article titles containing combinations of the keywords “Serra dos Órgãos,” “bryophytes,” “mosses,” and “liverworts,” followed by a careful and detailed reading of each selected publication; (ii) herbarium specimens collected in the study area; and (iii) new collections (Figure 4).

Herbarium specimens were obtained from online sources like (GBIF [81], jabot, JStorTypes and SpeciesLink accessed several times). The specimens are requested on loan and re-determined and carefully examined for small species associations, with one of each species cited as voucher in the Table 1. The authors conducted four field excursions to the PARNASO, each lasting one week, two during the rainy season and two during the dry season, with the aim of locating species not previously recorded in the herbarium. The collection, preservation and herborization methodologies follow Frahm (2003) and employ free walks [82] for covering all available substrates like tree trunks, logs, soil, rocks and leaves.

Field trips in the Park intended to check if species collected in the past are yet present, to collect new specimens and to record new taxa. Collected samples were deposited in the herbarium of the University of Brasília (UB), with duplicates sent to the herbarium of Instituto de Pesquisas Ambientais (SP). The classification system used here follows Crandall-Stotler et al. [83] for Marchantiophyta, Goffinet et al. [65] for Bryophyta and Renzaglia et al. [84] for Anthocerotophyta. Data referring to endemic species of Brazil and of Rio de Janeiro State came from Flora e Funga do Brasil (floradobrasil.jbrj.gov.br), while conservation status followed CNCFlora (cncflora.jbrj.gov.br).

## 5. Conclusions

The Serra dos Órgãos National Park stands out as a key refuge for bryophyte diversity in the Atlantic Forest, hosting numerous endemic and threatened species. The park’s altitudinal variation, diverse microhabitats, and unique environmental conditions favor high species richness and endemism. These findings reinforce the ecological value of PARNASO and underscore the urgency of conserving this unique mountainous ecosystem.

## Figures and Tables

**Table 1:** Richness and diversity of bryophyte species. Symbols on the species voucher: species only known from the literature (*).

Group	Family	Taxon	Voucher
**Liverworts**	Acrobolbaceae	*Tylimanthus laxus* (Lehm. & Lindenb.) Spruce	Peralta 27761 (SP)
	Aneuraceae	*Aneura pinguis* (L.) Dumort.	Peralta 27768 (SP)
	Aneuraceae	*Riccardia chamedryfolia* (With.) Grolle	Silva, AL 515
	Aneuraceae	*Riccardia digitiloba* (Spruce ex Steph.) Pagán	Peralta 27496 (SP)
	Aneuraceae	*Riccardia emarginata* (Steph.) Hell	Peralta 27618 (SP)
	Aneuraceae	*Riccardia fucoidea* (Sw.) Schiffn.	Peralta 20395 (SP)
	Aneuraceae	*Riccardia glaziou* (Spruce) Meenks	Gaspar 121 (R)
	Aneuraceae	*Riccardia regnellii* (Aongström.) Hell	Peralta 27487 (SP)
	Arnelliaceae	*Gongylanthus liebmannianus* (Lindenb. & Gott.) Steph	Gonçalves 358 (SP)
	Arnelliaceae	*Southbya organensis* Herzog *	Ph. v. Lützelburg 6003 p.p. (Tipo JE)
	Balantiopsidaceae	*Isotachis aubertii* (Schwägr.) Mitt.	Gaspar 148 (R)
	Balantiopsidaceae	*Isotachis erythrorhiza* (Lehm. & Lindenb.) Steph.	Peralta 27572 (SP)
	Balantiopsidaceae	*Isotachis multiceps* (Lindenb. & Gottsche) Gottsche	Lima 783 (SP)
	Balantiopsidaceae	*Isotachis serrulata* (Sw.) Gottsche	Gonçalves 396 (SP)
	Balantiopsidaceae	*Neesioscyphus carneus* (Nees) Grolle	Peralta 20398 (SP)
	Balantiopsidaceae	*Neesioscyphus homophyllus* (Nees) Grolle	Peralta 27722 (SP)
	Calypogeiaceae	*Calypogeia peruviana* Nees & Mont.	Peralta 27491 (SP)
	Cephaloziaceae	*Cephaloziella divaricada* (Sm.) Schiffn.	Gaspar 112 (R)
	Cephaloziaceae	*Cephaloziella granatensis* (J.B.Jack) Fulford	Peralta 27638 (SP)
	Cephaloziaceae	*Cylindrocolea planifolia* (Steph.) R.M.Schust.	Peralta 27621 (SP)
	Cephaloziaceae	*Kymatocalyx dominicensis* (Spruce) Vána	Peralta 27755 (SP)
	Cephaloziaceae	*Odontoschisma denudatum* (Nees) Dumort.	Peralta 20506 (SP)
	Cephaloziaceae	*Odontoschisma longiflorum* (Taylor) Trevis. *	Fulford (1976)
	Cephaloziellaceae	*Cephaloziopsis intertexta* (Gottsche) R.M. Schust.	Peralta 27488 (SP)
	Cephaloziellaceae	*Fuscocephaloziopsis crassifolia* (Lindenb. & Gottsche) Váňa & L. Söderstr.	Dias 1414 (SP)
	Dumortieraceae	*Dumortiera hirsuta* Nees & Mont.	Peralta 27710 (SP)
	Frullaniaceae	*Frullania atrata* Nees	Faria 1560 (SP)
	Frullaniaceae	*Frullania beyrichiana* (Lehm. & Lindenb.) Lehm. * & Lindenb.	Beyrich s.n. (holótipo W)
	Frullaniaceae	*Frullania brasiliensis* Raddi	Peralta 27631 (SP)
	Frullaniaceae	*Frullania cuencensis* Taylor	Peralta 27551 (SP)
	Frullaniaceae	*Frullania ericoides* (Nees) Mont. *	Nees (1833, as Jungermannia)
	Frullaniaceae	*Frullania kunzei* (Lehm. & Lindb.) Lehm. & Lindb.	Peralta 20492 (SP)
	Frullaniaceae	*Frullania obcordata* (Lehm. & Lindenb.) Lehm. & Lindenb. in Gott. et al. *	Winter & Schaefer-Verwimp (2020) [25]
	Frullaniaceae	*Frullania obscura* (Sw.) Dumort.	Peralta 20453 (SP)
	Frullaniaceae	*Frullania riojaneirensis* (Raddi) Spruce *	Gottsche et al. (1844) [26]
	Frullaniaceae	*Frullania schaefer-verwimpii* Yuzawa & Hatt.	Schafer-Verwimp 7375 (SP)
	Frullaniaceae	*Frullania setigera* Steph.	Peralta 27501 (SP)
	Frullaniaceae	*Frullania vitalii* Yuzawa & Hatt.	Yano 13575 (SP)
	Geocalycaceae	*Saccogynidium caldense* (Ångstr.) Grolle	Peralta 27620 (SP)
	Herbertaceae	*Herbertus acanthelius* Spruce	Peralta 27534 (SP)
	Herbertaceae	*Herbertus juniperoideus* (Sw.) Grolle	Gissi 339 (SP)
	Herbertaceae	*Herbertus pensilis* (T. Taylor) Spruce	Peralta 27612 (SP)
	Herbertaceae	*Herbertus sendtneri* (Nees) A.Evans *	Fulford (1963 as Herbertus grossispinus) [27]
	Herbertaceae	*Triandrophyllum subtrifidum* (Hook.f. & Taylor) Fulford & Hatch. *	Grolle (1964) [28]
	Jamesoniellaceae	*Syzygiella anomala* (Lindenb. & Gottsche) Steph.	Lima 789 p.p. (SP)
	Jamesoniellaceae	*Syzygiella colorata* (Lehm.) K. Feldberg et al.	Peralta 27575 (SP)
	Jamesoniellaceae	*Syzygiella concreta* (Gottsche) Spruce	Peralta 20397 (SP)
	Jamesoniellaceae	*Syzygiella contigua* Steph.	Peralta 20509 (SP)
	Jamesoniellaceae	*Syzygiella manca* (Mont.) J. B. Jack & Steph.	Gonçalves 389 (SP)
	Jamesoniellaceae	*Syzygiella perfoliata* (Sw.) Spruce	Peralta 20401 (SP)
	Jamesoniellaceae	*Syzygiella sonderi* (Gottsche) K. Feldberg, Váňa, Hentschel & Heinrichs	Peralta 27502 (SP)
	Jamesoniellaceae	*Syzygiella trigonifolia* (Steph.) Herzog	Peralta 27613 (SP)
	Jamesoniellaceae	*Syzygiella uleana* Steph.	Peralta 20428 (SP)
	Jungermanniaceae	*Anastrophyllum piligerum* (Nees) Steph.	Peralta 20380 (SP)
	Jungermanniaceae	*Anastrophyllum tubulosum* (Nees) Grolle	Peralta 27565 (SP)
	Jungermanniaceae	*Jungermannia amoena* Lindenb. & Gottsche	Gaspar 131 (R)
	Jungermanniaceae	*Jungermannia hyalina* Lyell	Lima 792 (SP)
	Jungermanniaceae	*Jungermannia sphaerocarpa* Hook.	Gaspar 122 (R)
	Lejeuneaceae	*Acanthocoleus aberrans* (Lindenb. & Gottsche) Kruijt	Peralta 27465 (SP)
	Lejeuneaceae	*Anoplolejeunea conferta* (C.F.W.Meissn. ex Spreng.) A.Evans	Peralta 27645 (SP)
	Lejeuneaceae	*Blepharolejeunea incongrua* (Lindenb. & Gottsche) van Slageren & Kruijt	Peralta 27577 (SP)
	Lejeuneaceae	*Brachiolejeunea phyllorhiza* (Nees) Kruijt & Gradst. *	Gottsche et al. (1844)
	Lejeuneaceae	*Bryopteris diffusa* (Sw.) Nees *	Stotler & Crandall (1974) [29]
	Lejeuneaceae	*Bryopteris filicina* (Sw.) Nees	Peralta 27530 (SP)
	Lejeuneaceae	*Ceratolejeunea rubiginosa* Gottsche ex Steph.	Peralta 27681 (SP)
	Lejeuneaceae	*Cheilolejeunea acutangula* (Nees) Grolle	Peralta 27523 (SP)
	Lejeuneaceae	*Cheilolejeunea beyrichii* (Lindenb.) E. Reiner	Peralta 27492 (SP)
	Lejeuneaceae	*Cheilolejeunea filiformis* (Sw.) W. Ye, R.L. Zhu & Gradst. *	Gottsche et al. (1844, as *Omphalanthus*) [26]
	Lejeuneaceae	*Cheilolejeunea grosseoleosa* C.J.Bastos & Schäf.-Verw.	Peralta 27549 p.p. (SP)
	Lejeuneaceae	*Cheilolejeunea holostipa* (Spruce) Grolle & R.-L.Zhu	Alkimim-Faria 1486
	Lejeuneaceae	*Cheilolejeunea unciloba* (Lindenb.) Malombe	Lima 750 (SP)
	Lejeuneaceae	*Cheilolejeunea xanthocarpa* (Lehm. & Lindenb.) Malombe	Peralta 27504 (SP)
	Lejeuneaceae	*Cololejeunea cardiocarpa* (Mont.) A.Evans	Peralta 27687 p.p. (SP)
	Lejeuneaceae	*Cololejeunea diaphana* A.Evans	Peralta 27687 p.p. (SP)
	Lejeuneaceae	*Cololejeunea jamesii* M.E.Reiner & Pócs	Peralta 20511 p.p. (SP)
	Lejeuneaceae	*Cololejeunea microscopica* (Taylor) Schiffn.	Peralta 27694 (SP)
	Lejeuneaceae	*Cololejeunea papilliloba* (Steph). Steph.	Peralta 27695 (SP)
	Lejeuneaceae	*Colura itatyana* Steph.	Gaspar 138 (R)
	Lejeuneaceae	*Colura tenuicornis* (A.Evans) Steph. *	Jovet-Ast (1953) [30]
	Lejeuneaceae	*Cyclolejeunea luteola* (Spruce) Grolle & R.-L.Zhu	Schafer-Verwimp 7416 (SP)
	Lejeuneaceae	*Dibrachiella parviflora* (Nees) X.Q. Shi, R.L. Zhu & Gradst.	Silva, AL 500
	Lejeuneaceae	*Dicranolejeunea axilaris* (Nees & Mont.) Schiffn.	Peralta 27735 (SP)
	Lejeuneaceae	*Diplasiolejeunea alata* Jovet-Ast	Peralta 27593 p.p. (SP)
	Lejeuneaceae	*Diplasiolejeunea unidentata* (Lehm. & Lindenb.) Schiffn. *	Gradstein & Costa (2003) [31]
	Lejeuneaceae	*Drepanolejeunea anoplanta* (Spruce) Steph.	Peralta 27659 (SP)
	Lejeuneaceae	*Drepanolejeunea araucariae* Steph.	Gaspar 135 (R)
	Lejeuneaceae	*Drepanolejeunea bidens* (Steph.) A.Evans	Gaspar 117 (R)
	Lejeuneaceae	*Drepanolejeunea fragilis* Bischl.	Peralta 27593 p.p. (SP)
	Lejeuneaceae	*Drepanolejeunea lichenicola* (Spruce) Steph. *	Bischler (1964) [32]
	Lejeuneaceae	*Drepanolejeunea mosenii* (Steph.) Bischl.	Peralta 27639 (SP)
	Lejeuneaceae	*Frullanoides tristis* van Slageren	Peralta 27646 (SP)
	Lejeuneaceae	*Haplolejeunea umbrosa* Gradst. & Ilkiu-Borges	Peralta 27495 (SP)
	Lejeuneaceae	*Harpalejeunea oxyphylla* (Nees & Mont.) Steph.	Gaspar 124 (R)
	Lejeuneaceae	*Harpalejeunea schiffneri* S.W. Arnell	Peralta 27462 (SP)
	Lejeuneaceae	*Harpalejeunea stricta* (Lindenb. & Gottsche) Steph.	Peralta 27526 (SP)
	Lejeuneaceae	*Harpalejeunea subacuta* A.Evans	Peralta 20450 p.p. (SP)
	Lejeuneaceae	*Lejeunea acanthogona* Spruce	Peralta 27463 (SP)
	Lejeuneaceae	*Lejeunea aphanes* Spruce	Lima 750 (SP)
	Lejeuneaceae	*Lejeunea bermudiana* (A.Evans) R.M.Schust.	Peralta 27719 (SP)
	Lejeuneaceae	*Lejeunea beyrichiana* (Steph) Gradst. & C.J.Bastos *	Beyrich s.n. (Tipo NY)
	Lejeuneaceae	*Lejeunea cerina* (Lehm. & Lindenb.) Gottsche	Schaefer-Verwimp 7369 (SP)
	Lejeuneaceae	*Lejeunea flaccida* Lindenb. & Gottsche	Peralta 27512 (SP)
	Lejeuneaceae	*Lejeunea flava* (Sw.) Nees	Peralta 27505 (SP)
	Lejeuneaceae	*Lejeunea glaucescens* Gottsche	Peralta 27476 (SP)
	Lejeuneaceae	*Lejeunea grossitexta* (Steph.) E.Reiner & Goda *	Schäfer-Verwimp & Vital (1989) [33]
	Lejeuneaceae	*Lejeunea laetevirens* Nees & Mont.	Peralta 27464 (SP)
	Lejeuneaceae	*Lejeunea longidentata* C.J.Bastos, Gradst., S. Vilas Bôas-Bastos & Schäf.-Verw.	Peralta 27543 (SP)
	Lejeuneaceae	*Lejeunea oligoclada* Spruce	Peralta 27498 p.p. (SP)
	Lejeuneaceae	*Lejeunea pterigonia* (Lehm. & Lindenb.) Mont.	Peralta 27525 (SP)
	Lejeuneaceae	*Lejeunea raddiana* Lindenb.	Peratla 20484 (SP)
	Lejeuneaceae	*Lejeunea serpillifolioides* (Raddi) Gradst.	Peralta 27611 (SP)
	Lejeuneaceae	*Lejeunea subplana* (Steph.) C.J.Bastos	Gonçalves 306 p.p. (SP)
	Lejeuneaceae	*Marchesinia brachiata* (Sw.) Schiffn.	Peralta 27508 (SP)
	Lejeuneaceae	*Metalejeunea cuculata* (Reinw. et al.) Grolle	Yano 13558 (SP)
	Lejeuneaceae	*Microlejeunea cystifera* Herzog	Peralta 27482 (SP)
	Lejeuneaceae	*Microlejeunea epiphylla* Bischl.	Gonçalves 306 p.p. (SP)
	Lejeuneaceae	*Microlejeunea squarrosa* J. Heinrichs, A. Schäfer-Verwimp, Pócs & S.S. Dong	Peralta 27671 p.p. (SP)
	Lejeuneaceae	*Microlejeunea stricta* (Lindenb. & Gottsche) Steph.	Lima 782 p.p. (SP)
	Lejeuneaceae	*Neurolejeunea breutelii* (Gottsche) A.Evans	Lima 767 p.p. (SP)
	Lejeuneaceae	*Otigoniolejeunea huctumalcensis* (Lindenb. & Gottsche) Y.M. Wei, R.L. Zhu & Gradst.	Peralta 27474 (SP)
	Lejeuneaceae	*Prionolejeunea aemula* (Gottsche) A.Evans	Peralta 27727 (SP)
	Lejeuneaceae	*Schiffneriolejeunea polycarpa* (Nees) Gradst.	Peralta 20468 (SP)
	Lejeuneaceae	*Vitalianthus bischelerianus* (Porto & Grolle) R.M.Schust. & Giancotti *	Gottsche et al. (1844, as Phragmicoma juliformis)
	Lepidoziaceae	*Bazzania aurescens* Spruce	Peralta 20394 (SP)
	Lepidoziaceae	*Bazzania hookeri* (Lindenb.) Trevis.	Fulford (1963)
	Lepidoziaceae	*Bazzania longistipula* (Lindenb.) Trevis.	Peralta 27480 (SP)
	Lepidoziaceae	*Bazzania nitida* (Weber) Grolle	Peralta 27529 (SP)
	Lepidoziaceae	*Bazzania stolonifera* (Sw.) Trevis. *	Nees (1833, as Jungermannia) [34]
	Lepidoziaceae	*Kurzia capillaris* (Sw.) Grolle	Lima 780 (SP)
	Lepidoziaceae	*Lepidozia coilophylla* Taylor	Peralta 27532 (SP)
	Lepidoziaceae	*Lepidozia cupressina* (Sw.) Lindenb.	Gonçalves 311 (SP)
	Lepidoziaceae	*Lepidozia inaequalis* (Lehm. & Lindenb.) Lehm. & Lindenb.	Peralta 20402 (SP)
	Lepidoziaceae	*Lepidozia incurvata* Lindenb.	Lima 784 (SP)
	Lepidoziaceae	*Lepidozia macrocolea* Spruce	Peralta 27539 (SP)
	Lepidoziaceae	*Micropterygium leiophyllum* Spruce *	Gottsche et al. (1844, as M. vulgare)
	Lepidoziaceae	*Micropterygium pterygophyllum* (Nees) Trevis.	Nees (1833, as Jungermannia)
	Lepidoziaceae	*Paracromastigum dusenii* R.M.Schust. *	Dias 1310 (SP)
	Lepidoziaceae	*Paracromastigum pachyrhizum* (Nees) Fulford	Schafer-Verwimp 7421 (SP)
	Lepidoziaceae	*Telaranea nematodes* (Gottsche ex Austin) M.A.Howe	Lima 754 (SP)
	Lophocoleaceae	*Lophocolea muricata* (Lehm.) Nees in Gottsche et al.	Peralta 27508 (SP)
	Lophocoleaceae	*Clasmatocolea vermicularis* (Lehm.) Grolle	Peralta 27566 (SP)
	Lophocoleaceae	*Cryptolophocolea martiana* (Nees) L.Soderstr. et al. subsp. martiana	Lima 768 (SP)
	Lophocoleaceae	*Heteroscyphus combinatus* (Nees) Schiffn.	Peralta 27556 (SP)
	Lophocoleaceae	*Leptoscyphus amphibolius* (Nees) Grolle	Peralta 27583 (SP)
	Lophocoleaceae	*Leptoscyphus porphyrius* (Nees) Grolle	Peralta 27628 (SP)
	Lophocoleaceae	*Leptoscyphus spectabilis* (Steph.) Grolle	Peralta 27658 (SP)
	Lophocoleaceae	*Lophocolea bidentata* (L.) Dumort.	Peralta 20420 (SP)
	Lophocoleaceae	*Lophocolea connata* (Sw.) Nees *	Nees (1833, as Jungermannia)
	Lophocoleaceae	*Lophocolea mandonii* Steph.	Peralta 20379 (SP)
	Marchantiaceae	*Marchantia chenopoda* L.	Vital 1197 (SP)
	Marchantiaceae	*Marchantia polymorpha* L.	Schwacke, C.A.W. s.n. (R)
	Marchantiaceae	*Marchantia paleacea* Bert.	Peralta 20475 (SP)
	Metzgeriaceae	*Metzgeria acuminata* Steph.	Peralta 27471 (SP)
	Metzgeriaceae	*Metzgeria albinea* Spruce	Peralta 27475 (SP)
	Metzgeriaceae	*Metzgeria brasiliensis* Schiffn.	Dias 1234 (SP)
	Metzgeriaceae	*Metzgeria ciliata* Raddi *	Raddi (1823) [35]
	Metzgeriaceae	*Metzgeria conjugata* Lindb.	Silva, AL 517 (UB)
	Metzgeriaceae	*Metzgeria consanguinea* Schiffn.	Peralta 27528 (SP)
	Metzgeriaceae	*Metzgeria convoluta* Steph. *	Costa (2008) [36]
	Metzgeriaceae	*Metzgeria cratoneura* Schiffn. *	Costa (2008) [36]
	Metzgeriaceae	*Metzgeria decipiens* (C. Massal.) Schiffn.	Eiten 7105 (SP)
	Metzgeriaceae	*Metzgeria fruticola* Spruce	Peralta 27715 (SP)
	Metzgeriaceae	*Metzgeria furcata* (L.) Dumort. *	Bartram (1954) Gonçalves 326 (SP)
	Metzgeriaceae	*Metzgeria hegewaldii* Kuwah.	Peralta 27466 (SP)
	Metzgeriaceae	*Metzgeria lechlerii* Steph. *	Costa et al. (2005) [37]
	Metzgeriaceae	*Metzgeria myriopoda* Lindb.	Yano 13561 (SP)
	Noterocladaceae	*Noteroclada confluens* (Hook.f. & Taylor) Spruce	Peralta 20471 (SP)
	Pallaviciniaceae	*Jensenia spinosa* (Lindenb. & Gottsche) Grolle	Gonçalves 400 (SP)
	Pallaviciniaceae	*Pallavicinia lyelii* (Hook.) S.F.Gray	Gonçalves 186 (SP)
	Pallaviciniaceae	*Symphyogyna brasiliensis* (Nees) Nees & Mont.	Peralta 27750 (SP)
	Pallaviciniaceae	*Symphyogyna podophylla* (Thunb.) Mont. & Nees	Silva, AL 502
	Plagiochilaceae	*Plagiochila amicta* Steph. *	Heinrichs (2002) [38]
	Plagiochilaceae	*Plagiochila bicuspidata* Gottsche	Peralta 10518 (SP)
	Plagiochilaceae	*Plagiochila bifaria* (Sw.) Lindenb.	Peralta 27516 (SP)
	Plagiochilaceae	*Plagiochila boryana* Gottsche ex Steph.	Peralta 27749 (SP)
	Plagiochilaceae	*Plagiochila corrugata* (Nees) Nees & Mont.	Peralta 20376 (SP)
	Plagiochilaceae	*Plagiochila crispabilis* Lindenb.	Peralta 20511 (SP)
	Plagiochilaceae	*Plagiochila cristata* (Sw.) Lindenb.	Schafer-Verwimp 7401 (SP)
	Plagiochilaceae	*Plagiochila deflexirama* Taylor	Peralta 27721 (SP)
	Plagiochilaceae	*Plagiochila exigua* (Taylor) Taylor	Peralta 20468 (SP)
	Plagiochilaceae	*Plagiochila flabelliflora* Steph.	Gonçalves 334 (SP)
	Plagiochilaceae	*Plagiochila gymnocalycina* (Lehm. & Lindenb.) Lindenb.	Dias 1397 (SP)
	Plagiochilaceae	*Plagiochila loriloba* Herzog ex Carl	Perlata 27519 (SP)
	Plagiochilaceae	*Plagiochila macrotachya* Lindenb.	Peralta 27700 (SP)
	Plagiochilaceae	*Plagiochila martiana* (Nees) Lindenb.	Peralta 20385 (SP)
	Plagiochilaceae	*Plagiochila montagnei* Nees & Mont. *	Heinrichs & Gradstein (2000) [39]
	Plagiochilaceae	*Plagiochila pseudoradicans* Herzog *	Ph. v. Lützelburg 6029b (type JE)
	Plagiochilaceae	*Plagiochila punctata* (Taylor) Taylor	Lima 783 p.p. (SP)
	Plagiochilaceae	*Plagiochila rutilans* Lindenb. *	Heinrichs (2002) [38]
	Plagiochilaceae	*Plagiochila simplex* (Sw.) Lindenb.	Peralta 27780 (SP)
	Plagiochilaceae	*Plagiochila subbidentata* Taylor	Peralta 27586 (SP)
	Plagiochilaceae	*Plagiochila superba* d’Orbigny	Peralta 20457 (SP)
	Porellaceae	*Porella brasiliensis* (Raddi) Schiffn.	Peralta 27724 (SP)
	Porellaceae	*Porella reflexa* (Lehm. & Lindenb.) Trevis.	Yano 13580 (SP)
	Radulaceae	*Radula angulata* Steph.	Peralta 27697 (SP)
	Radulaceae	*Radula javanica* Gottsche	Peralta 27527 (SP)
	Radulaceae	*Radula ligula* Steph.	Peralta 20407 (SP)
	Radulaceae	*Radula nudicaulis* Steph.	Gonçalves 408 (SP)
	Radulaceae	*Radula pallens* (Sw.) Nees ex Mont	Lima 759 (SP)
	Radulaceae	*Radula recubans* Taylor	Gaspar 142 (R)
	Radulaceae	*Radula schaefer-verwimpii* K.Yamada	Peralta 27489 (SP)
	Radulaceae	*Radula stenocalyx* Mont.	Peralta 27691 (SP)
	Radulaceae	*Radula subinflata* Lindenb. & Gottsche	Gonçalves 322 (SP)
	Radulaceae	*Radula tectiloba* Steph.	Peralta 27507 (SP)
	Radulaceae	*Radula voluta* Taylor ex Gottsche, Lindenb. & Nees	Peralta 20429 (SP)
	Scapaniaceae	*Scapania portoricensis* Hampe & Gottsche	Peralta 27541 (SP)
	Trichocoleaceae	*Trichocolea brevifissa* Steph.	Peralta 27503 (SP)
**Mosses**	Adelotheciaceae	*Adelothecium bogotense* (Hampe) Mitt.	Peralta 27692 (SP)
	Andreaceae	*Andreaea acutifolia* Hook.f. & Wilson *	Müller (1845) Gonçalves 401 (SP)
	Andreaceae	*Andreaea rupestris* Hedw.	Gaspar 137 (R)
	Andreaceae	*Andreaea subulata* Harv. *	Lützelburg, P. von 7041 (tipo JE of Andreaea hamulata)
	Bartramiaceae	*Bartramia mathewsii* Mitt.	Peralta 27606 (SP)
	Bartramiaceae	*Breutelia grandis* (Hampe) Paris	Peralta 27559 (SP)
	Bartramiaceae	*Breutelia tomentosa* (Sw. ex Brid.) A.Jaeger	Lima 798 (SP)
	Bartramiaceae	*Leiomela bartramioides* (Hook.) Paris	Peralta 27500 (SP)
	Bartramiaceae	*Leiomela piligera* (Hampe) Broth. *	Brotherus (1924) [40]
	Bartramiaceae	*Philonotis cernua* (Wilson) Griffin & W.R.Buck	Peralta 20426 (SP)
	Bartramiaceae	*Philonotis hastata* (Duby) Wijk & Margad.	Alkimim-Faria, 1513
	Bartramiaceae	*Philonotis uncinata* (Schwägr.) Brid. *	Brotherus (1894)
	Brachytheciaceae	*Aerolindigia capillacea* (Hornsch.) M.Menzel	Peralta 20418 (SP)
	Brachytheciaceae	*Meteoridium remotifolium* (Müll.Hal.) Manuel	Peralta 27763 (SP)
	Brachytheciaceae	*Rhynchostegium scariosum* (Taylor) A.Jaeger	Peralta 20419 (SP)
	Brachytheciaceae	*Rhynchostegium serrulatum* (Hedw.) A.Jaeger	Gonçalves 333 p.p. (SP)
	Brachytheciaceae	*Squamidium brasiliense* Broth. *	Allen & Crosby (1986) [41]
	Brachytheciaceae	*Squamidium leucotrichum* (Taylor) Broth.	Peralta 27486 (SP)
	Brachytheciaceae	*Zelometeorium ambiguum* (Hornsch.) Manuel *	Manuel (1977) [42]
	Brachytheciaceae	*Zelometeorium patulum* (Hedw.) Manuel	Gonçalves 332 (SP)
	Bruchiaceae	*Trematodon longicollis* Michx. *	Brotherus (1924b) [43]
	Bryaceae	*Anomobryum julaceum* (Schrad. ex P.Gaertn. et al.) Schimp.	Peralta 20377 (SP)
	Bryaceae	*Brachymenium acuminatum* Harv.	Dias 1372 (SP)
	Bryaceae	*Brachymenium consimile* (Mitt.) A.Jaeger	Peralta 27654 (SP)
	Bryaceae	*Brachymenium hornschuchianum* Mart. *	Brotherus (1894) [44]
	Bryaceae	*Brachymenium radiculosum* (Schwägr.) Hampe *	Brotherus (1924b) [43]
	Bryaceae	*Brachymenium systilium* (Müll.Hal.) A.Jaeger	Peralta 27673 (SP)
	Bryaceae	*Bryum argenteum* Hedw.	Vital 1191 (SP)
	Bryaceae	*Bryum atenense* Williams	Alkimim-Faria 1246 (UB)
	Bryaceae	*Bryum billarderii* Schwaegr.	Dias 1306a (SP)
	Bryaceae	*Bryum capillare* Hedw.	Peralta 20465 (SP)
	Bryaceae	*Bryum densifolium* Brid. *	Bartram (1954, as *B. gracilescens*)
	Bryaceae	*Bryum huillense* Welw. & Duby	Peralta 20415 (SP)
	Bryaceae	*Bryum limbatum* Müll.Hal *	Gonçalves 384 (SP)
	Bryaceae	*Bryum pabstianum* Müll.Hal. *	Hampe (1879) [45]
	Bryaceae	*Bryum subapiculatum* Hampe	Peralta 20462 (SP)
	Bryaceae	*Rhodobryum aubertii* (Schwägr.) Thér.	Peralta 27762 (SP)
	Bryaceae	*Rhodobryum beyrichianum* (Hornsch.) Müll.Hal.	Peralta 20514 (SP)
	Bryaceae	*Rhodobryum roseum* (Hedw.) Limpr.	Peralta 27582 (SP)
	Bryaceae	*Rhodobryum subverticillatum* Broth. *	Bartram (1952) [46]
	Bryaceae	*Schoenobryum concavifolium* (Griff.) Gangulee *	Bartram (1954) [19]
	Calymperaceae	*Calymperes nicaraguense* Renauld & Cardot	Alkimim-Faria 1256
	Calymperaceae	*Syrrhopodon gaudichaudii* Mont. *	Bartram (1954) [19]
	Calymperaceae	*Syrrhopodon helicophyllus* Mitt.	Alkimim-Faria, AL 1254
	Calymperaceae	*Syrrhopodon ligulatus* Mont.	Peralta 27490 (SP)
	Calymperaceae	*Syrrhopodon prolifer* Schwägr.	Peralta 27473 (SP)
	Catagoniaceae	*Catagonium brevicaudatum* Müll.Hal. ex Broth.	Peralta 27630 (SP)
	Daltoniaceae	*Calyptrochaeta albescens* (Hampe) W.R.Buck	Lima 775 (SP)
	Daltoniaceae	*Calyptrochaeta setigera* (Mitt.) W.R.Buck	Peralta 27702 (SP)
	Daltoniaceae	*Daltonia splachnoides* (Sm.) Hook. & Taylor	Peralta 27546 (SP)
	Daltoniaceae	*Leskeodon aristatus* (Geh. & Hampe) Broth.	Peralta 27517 (SP)
	Dicranaceae	*Aongstroemia julacea* (Hook.) Mitt.	Peralta 27550 (SP)
	Dicranaceae	*Atractylocarpus brasiliensis* (Müll.Hal.) R.S.Williams	Peralta 20490 (SP)
	Dicranaceae	*Atractylocarpus longisetus* (Hook.) E.B.Bartram *	Bartram (1954) [19]
	Dicranaceae	*Dicranella harrisii* Müll.Hal.) Broth.	Schafer-Verwimp 13101 (SP)
	Dicranaceae	*Dicranella hilariana* (Mont.) Mitt. *	Luetzelburg (1923, as *D. trematodontifolia*) [47]
	Dicranaceae	*Dicranella subsulcata* (Hampe) Hampe	Schafer-Verwimp 7422 (SP)
	Dicranaceae	*Dicranodontium pulchroalare* Broth. *	Lützelburg 1519 (holótipo JE)
	Dicranaceae	*Dicranoloma billardieri* (Brid. ex Anon) Paris	Peralta 27597 (SP)
	Dicranaceae	*Dicranum frigidum* Müll.Hal.	Dias 1361 (SP)
	Dicranaceae	*Holomitrium crispulum* Mart.	Peralta 27599 (SP)
	Dicranaceae	*Leucoloma cruegerianum* (Müll.Hal.) A.Jaeger	Peralta 27678 (SP)
	Dicranaceae	*Leucoloma serrulatum* Brid.	Peralta 27701 (SP)
	Dicranaceae	*Leucoloma triforme* (Mitt.) A.Jaeger	Gonçalves 190 (SP)
	Dicranaceae	*Microcampylopus curvisetus* (Hampe) Giese & J.-P.Frahm	Schafer-Verwimp 7422 p.p. (SP)
	Dicranaceae	*Paraleucobryum longifolium* (Hedw.) Loeske	Dias 1325 (SP)
	Dicranaceae	*Pilopogon guadalupensis* (Brid.) J.-P.Frahm	Peralta 1558 (SP)
	Diphysciaceae	*Diphyscium longifolium* Griff.	Peralta 27723 (SP)
	Ditrichaceae	*Ceratodon purpureus* (Hedw.) Brid.	Dias 1315 (SP)
	Ditrichaceae	*Cladastomum robustum* Broth.	Lima 485 (SP)
	Ditrichaceae	*Cladastomum ulei* Müll.Hal.	Peralta 27635 (SP)
	Ditrichaceae	*Crumuscus vitalis* W.R.Buck & Snider	Lima 791 (SP)
	Ditrichaceae	*Ditrichum crinale* (Taylor) Kuntze	Lima 801 (SP)
	Ditrichaceae	*Ditrichum paulense* Geh. ex Hampe	Peralta 27656 (SP)
	Entodontaceae	*Entodon hampeanus* Müll.Hal.	Peralta 27614 (SP)
	Entodontaceae	*Entodon jamesonii* (Taylor) Mitt.	Peralta 27580 (SP)
	Entodontaceae	*Erythrodontium longisetum* (Hook.) Paris *	Vattimo-Gil & Vattimo (1980) [48]
	Entodontaceae	*Erythodontium squarrosum* (Hampe) Paris *	Brotherus (1924)
	Ephemeraceae	*Micromitrium tenerum* (Bruch & Schimp.) Crosby	Alkimim-Faria, AL 1379 (UB)
	Fabroniaceae	*Dimerodontium pellucidum* Schwägr.	Schafer-Verwimp 13113 (SP)
	Fabroniaceae	*Fabronia ciliaris* (Brid.) Brid.	Gonçalves 182 (SP)
	Fissidentaceae	*Fissidens anguste-limbatus* Mitt.	Peralta 27665 (SP)
	Fissidentaceae	*Fissidens asplenioides* Hedw.	Peralta 27709 (SP)
	Fissidentaceae	*Fissidens bryoides* Hedw.	Lima 787 (SP)
	Fissidentaceae	*Fissidens crispus* Mont.	Gonçalves 180 (SP)
	Fissidentaceae	*Fissidens elegans* Brid.	Peralta 27479 (SP)
	Fissidentaceae	*Fissidens flabellatus* Hornsch. *	Beyrich s.n. (lectótipo BM)
	Fissidentaceae	*Fissidens intromarginatus* (Hampe) Mitt. *	Ule 1225 (lectotype H, tipo de F. hemibryoides)
	Fissidentaceae	*Fissidens lagenarius* Mitt. *	Bartram (1954)
	Fissidentaceae	*Fissidens oblongifolius* Hook.f. & Wilson	Peralta 20464 (SP)
	Fissidentaceae	*Fissidens oediloma* Müll.Hal. ex Broth.	Peralta 27571 (SP)
	Fissidentaceae	*Fissidens pellucidus* Hornsch.	Peralta 27538 (SP)
	Fissidentaceae	*Fissidens prionodes* Mont.	Peralta 27740 (SP)
	Fissidentaceae	*Fissidens radicans* Mont.	Peralta 27737 (SP)
	Fissidentaceae	*Fissidens rigidulus* Hook.f. & Wilson	Peralta 27756 (SP)
	Fissidentaceae	*Fissidens saprofilus* Broth.	Lima 754 (SP)
	Fissidentaceae	*Fissidens scariosus* Mitt.	Lima 756 (SP)
	Fissidentaceae	*Fissidens semicompletus* Hedw.	Alkimim-Faria 1228
	Fissidentaceae	*Fissidens taxifolius* Hedw.	Alkimim-Faria 1367
	Fissidentaceae	*Fissidens wallisii* Müll.Hal. ex Broth.	Peralta 27675 (SP)
	Fissidentaceae	*Fissidens weirii* Mitt.	Peralta 27484 (SP)
	Funariaceae	*Entosthodon bonplandii* (Hook.) Mitt.	Schaefer-Verwimp 7405 (SP)
	Funariaceae	*Funaria hygrometrica* Hedw. *	Müller (1845) [49]
	Grimmiaceae	*Grimmia laevigata* (Brid.) Brid.	Dias 1298 (SP)
	Grimmiaceae	*Grimmia longirostris* Hook.f. & Wilson	Gaspar 110 (R)
	Grimmiaceae	*Racomitrium subsecundum* (Hook. & Grev. ex Harv.) Mitt.	Dias 1405 (SP)
	Hedwigiaceae	*Hedwigidium rhabdocarpum* (Hampe) A.Jaeger	Faria 1547 (SP)
	Hookeriaceae	*Hookeria acutifolia* Hook. & Grev.	Peralta 27739 (SP)
	Hookeriaceae	*Philophyllum tenuifolium* (Mitt.) Broth.	Peralta 27581 (SP)
	Hypnaceae	*Chrysohypnum diminutivum* (Mitt.) Broth.	Peralta 20478 (SP)
	Hypnaceae	*Ectropothecium leptochaeton* (Schwägr.) W.R.Buck	Peralta 20461 (SP)
	Hypnaceae	*Mittenothamnium reduncum* (Mitt.) Ochyra	Peralta 27624 (SP)
	Hypnaceae	*Mittenothamnium reptans* (Hedw.) Cardot	Eiten 7133 (SP)
	Hypnaceae	*Taxiphyllum taxirameum* (Mitt.) M. Fleisch.	Gonçalves 194 (SP)
	Hypnaceae	*Vesicularia vesicularis* (Schwägr.) Broth.	Alkimim-Faria, 1364
	Hypopterygiaceae	*Hypopterygium tamariscinum* (Hedw.) Brid.	Yano 13560 (SP)
	Hypopterygiaceae	*Lopidium concinnum* (Hook.) Wilson	Peralta 27712 (SP)
	Lembophyllaceae	*Orthostichella versicolor* (Müll.Hal.) B.H.Allen & W.R.Buck	Peralta 27520 (SP)
	Leucobryaceae	*Campylopus aemulans* (Hampe) A.Jaeger *	Frahm (1975) [50]
	Leucobryaceae	*Campylopus arctocarpus* (Hornsch.) Mitt.	Peralta 27547 (SP)
	Leucobryaceae	*Campylopus cryptopodioides* Broth. *	Frahm (1991) [15]
	Leucobryaceae	*Campylopus cuspidatus* (Hornsch.) Mitt. *	Frahm (1975) [50]
	Leucobryaceae	*Campylopus densicoma* (Müll.Hal.) Paris	Gonçalves 409 (SP)
	Leucobryaceae	*Campylopus dichrostis* (Müll.Hal.) Paris	Gonçalves 330 (SP)
	Leucobryaceae	*Campylopus filifolius* (Müll.Hal.) Paris	Lima 789 (SP)
	Leucobryaceae	*Campylopus fragilis* (Brid.) Bruch & Schimp.	Peralta 27610 (SP)
	Leucobryaceae	*Campylopus gemmatus* (Müll.Hal.) Paris	Vital 7634 (SP)
	Leucobryaceae	*Campylopus heterostachys* (Hampe) A.Jaeger	Dias 1299 (SP)
	Leucobryaceae	*Campylopus introflexus* (Hedw.) Brid.	Alkimim-Faria 1510
	Leucobryaceae	*Campylopus julicaulis* Broth.	Gaspar 104 (R)
	Leucobryaceae	*Campylopus lamellinervis* (Müll.Hal.) Mitt.	Müller 1845
	Leucobryaceae	*Campylopus occultus* Mitt.	Frahm sn (SP147028)
	Leucobryaceae	*Campylopus pilifer* Brid.	Peralta 27605 (SP)
	Leucobryaceae	*Campylopus pyriformis* (Schultz) Brid.	Gaspar 114 (R)
	Leucobryaceae	*Campylopus richardii* Brid.	Dias 1324 (SP)
	Leucobryaceae	*Campylopus savannarum* (Müll.Hal.) Mitt.	Gaspar 139 (R)
	Leucobryaceae	*Campylopus subcuspidatus* (Hampe) A.Jaeger	Peralta 27641 (SP)
	Leucobryaceae	*Campylopus thwaitesii* (Mitt.) A.Jaeger	Gonçalves 183 (SP)
	Leucobryaceae	*Campylopus trachyblepharon* (Müll.Hal.) Mitt.	Paula sn (SP463831)
	Leucobryaceae	*Campylopus uleanus* (Müll.Hal.) Broth.	Dias 1384 (SP)
	Leucobryaceae	*Campylopus widgrenii* (Müll.Hal.) Mitt.	Vital 1189 (SP)
	Leucobryaceae	*Leucobryum albicans* (Schwägr.) Lindb.	Peralta 27522 (SP)
	Leucobryaceae	*Leucobryum clavatum* Hampe *	Luetzelburg 1923 [47]
	Leucobryaceae	*Leucobryum crispum* Müll.Hal.	Dias 1235 (SP)
	Leucobryaceae	*Leucobryum giganteum* Müll.Hal. *	Herzog (1926) [51]
	Leucobryaceae	*Leucobryum sordidum* Ångström *	Luetzelburg (1923) [47]
	Leucobryaceae	*Ochrobryum gardneri* (Müll.Hal.) Lindb.	Alkimim-Faria 1364 (UB)
	Leucobryaceae	*Octoblepharum albidum* Hedw. *	Bartram (1954) [19]
	Leucomiaceae	*Leucomium strumosum* (Hornsch.) Mitt.	Peralta 27769 (SP)
	Meteoriaceae	*Meteorium deppei* (Hornsch.) Mitt.	Peralta 27573 (SP)
	Meteoriaceae	*Meteorium nigrescens* (Hedw.) Dozy & Molk. *	Brotherus (1924)
	Meteoriaceae	*Meteorium pseudoteres* W.R.Buck	Peralta 27668 (SP)
	Meteoriaceae	*Pilotrichella flexilis* (Hedw.) Ångström	Peralta 20491 (SP)
	Meteoriaceae	*Toloxis imponderosa* (Taylor) W.R.Buck	Peralta 20517 (SP)
	Mniaceae	*Epipterygium immarginatum* Mitt.	Peralta 20432 (SP)
	Mniaceae	*Plagiomnium rhynchophorum* (Hook.) T.J.Kop.	Peralta 20411 p.p. (SP)
	Mniaceae	*Pohlia camptotrachela* (Renauld & Cardot) Broth.	Peralta 20433 (SP)
	Mniaceae	*Pohlia elongata* Hedw.	Lima 790 (SP)
	Mniaceae	*Pohlia papilosa* (Müll.Hal. ex A.Jaeger) Broth.	Faria 1554 (SP)
	Mniaceae	*Pohlia tenuifolia* (A.Jaeger) Broth.	Peralta 27579 (SP)
	Mniaceae	*Schizymenium campylocarpum* (Arn. & Hook.) A.J.Shaw	Lima 761 (SP)
	Mniaceae	*Schizymenium pusillum* (Hook.f. & Wilson) A.J.Shaw	Dias 1382 (SP)
	Neckeraceae	*Homalia glabella* (Hedw.) Schimp.	Gonçalves 310 (SP)
	Neckeraceae	*Homaliodendron flabellatum* (Sm.) M.Fleisch.	Peralta 27569 (SP)
	Neckeraceae	*Neckera ehrenbergii* Müll.Hal.	Peralta 27588 (SP)
	Neckeraceae	*Porotrichum korthalsianum* (Dozy & Molk.) Mitt.	Dias 1420 (SP)
	Neckeraceae	*Porotrichum leucocaulon* Müll.Hal.	Peralta 27674 (SP)
	Neckeraceae	*Porotrichum longirostre* (Hook.) Mitt.	Peralta 20387 p.p. (SP)
	Neckeraceae	*Porotrichum superbum* (Taylor) Hampe	Peralta 27585 (SP)
	Neckeraceae	*Porotrichum thieleanum* (Müll.Hal.) Mitt.	Peralta 20383 (SP)
	Neckeraceae	*Thamnobryum fasciculatum* (Hedw.) I.Sastre	Peralta 27703 (SP)
	Orthotrichaceae	*Macrocoma orthotrichoides* (Raddi) Wijk & Margad.	Schafer-Verwimp 7449 (SP)
	Orthotrichaceae	*Macromitrium catharinense* Paris *	Li et al. (2019) [52]
	Orthotrichaceae	*Macromitrium cirrosum* (Hedw.) Brid. *	Bartram (1954)
	Orthotrichaceae	*Macromitrium microstomum* (Hook. & Grev.) Schwägr.	Gonçalves 395 (SP)
	Orthotrichaceae	*Orthotrichum papillosum* (Hampe) Lesquereux & James	Alkimim-Faria, 1236
	Orthotrichaceae	*Schlotheimia appressifolia* Mitt.	Atwood 2007
	Orthotrichaceae	*Schlotheimia capillaris* Hampe	Peralta 20376 p.p. (SP)
	Orthotrichaceae	*Schlotheimia rugifolia* (Hook.) Schwägr.	Lima 753 (SP)
	Orthotrichaceae	*Schlotheimia tecta* Hook.f. & Wilson	Gonzatti 4003 (SP)
	Orthotrichaceae	*Zygodon reiwardtii* (Hornsch.) A.Braun	Peralta 27629 (SP)
	Phylogoniaceae	*Phyllogonium viride* Brid.	Gonçalves 320 (SP)
	Pilotrichaceae	*Callicostella martiana* (Hornsch.) A.Jaeger	Gonçalves 197 (SP)
	Pilotrichaceae	*Callicostella pallida* (Hornsch.) Ångström	Peralta 27689 (SP)
	Pilotrichaceae	*Cyclodictyon albicans* (Hedw.) Kuntze	Peralta 27587 (SP)
	Pilotrichaceae	*Cyclodictyon marginatum* (Hook. & Wilson) Kuntze	Schafer-Verwimp 13124 (SP)
	Pilotrichaceae	*Cyclodictyon varians* (Sull.) Kuntze	Peralta 27619 (SP)
	Pilotrichaceae	*Lepidopilidium brevisetum* (Hampe) Broth.	Silva, AL 481A (UB)
	Pilotrichaceae	*Lepidopilidium nitens* (Hornsch.) Broth.	Peralta 27764 (SP)
	Daltoniaceae	*Lepidopilum affine* Müll.Hal. ex Broth.	Alkimim-Faria, 1360 (UB)
	Pilotrichaceae	*Lepidopilum muelleri* (Hampe) Mitt.	Peralta 20409 (SP)
	Pilotrichaceae	*Lepidopilum scabrisetum* (Schwägr.) Steere	Peralta 27666 (SP)
	Pilotrichaceae	*Lepidopilum subsubulatum* Geh. & Hampe	Peralta 27521 (SP)
	Pilotrichaceae	*Thamniopsis incurva* (Hornsch.) W.R.Buck	Yano 13564 (SP)
	Pilotrichaceae	*Thamniopsis langsdorffii* (Hook.) W.R.Buck	Gonçalves 307 (SP)
	Pilotrichaceae	*Trachyxiphium guadalupense* (Brid.) W.R.Buck	Peralta 27497 p.p. (SP)
	Plagiotheciaceae	*Plagiothecium novogranatense* (Hampe) Mitt.	Peralta 27570 (SP)
	Polytrichaceae	*Atrichum androgynum* (Müll.Hal.) A.Jaeger	Gonçalves 195 (SP)
	Polytrichaceae	*Itatiella ulei* (Broth. ex Müll.Hal.) G.L.Sm.	Peralta 27634 (SP)
	Polytrichaceae	*Oligotrichum riedelianum* Mitt.	Peralta 27594 (SP)
	Polytrichaceae	*Pogonatum campylocarpum* (Müll.Hal.) Mitt.	Peralta 20439 (SP)
	Polytrichaceae	*Pogonatum pensilvanicum* (E.B.Bartram ex Hedw.) P.Beauv.	Paula, J.C. sn (SP463829)
	Polytrichaceae	*Pogonatum perichaetiale* (Mont.) A.Jaeger	Alkimim-Faria 1236 (UB)
	Polytrichaceae	*Polytrichadelphus pseudopolytrichum* (Raddi) G.L.Sm.	Eiten 7156 (SP)
	Polytrichaceae	*Polytrichum angustifolium* Mitt.	Peralta 20378 (SP)
	Polytrichaceae	*Polytrichum commune* L. ex Hedw.	Goeldi sn (SP219763)
	Polytrichaceae	*Polytrichum juniperinum* Willd. ex Hedw.	Faria 1562 (SP)
	Pottiaceae	*Barbula indica* (Hook.) Spreng.	Peralta 27774 (SP)
	Pottiaceae	*Chionoloma angustatum* (Mitt.) M.Menzel	Peralta 27677 (SP)
	Pottiaceae	*Chionoloma fractum* M.J.Cano, J.A.Jiménez & M.Alonso	Peralta 27589 (SP)
	Pottiaceae	*Hyophila involuta* (Hook.) A.Jaeger	Silva, AL 499 (UB)
	Pottiaceae	*Hyophiladelphus agrarius* (Hedw.) R.H.Zander *	Bartram (1954)
	Pottiaceae	*Leptodontium araucarieti* (Müll.Hal.) Paris	Gonçalves 413 (SP)
	Pottiaceae	*Leptodontium filicicola* Herzog	Peralta 27669 (SP)
	Pottiaceae	*Leptodontium flexifolium* (Dicks.) Hampe	Dias 1276 (SP)
	Pottiaceae	*Leptodontium pungens* (Mitt.) Kindb.	Dias 1377 (SP)
	Pottiaceae	*Leptodontium stellatifolium* (Hampe) Broth.	Peralta 27595 (SP)
	Pottiaceae	*Leptodontium viticulosoides* (P.Beauv.) Wijk & Margad.	Peralta 27607 (SP)
	Pottiaceae	*Leptodontium wallisii* (Müll.Hal.) Kindb.	Gonçalves 364 (SP)
	Pottiaceae	*Streptopogon calymperes* Müll.Hal.	Peralta 27450 (SP)
	Pottiaceae	*Tortella humilis* (Hedw.) Jenn.	Peralta 20417 (SP)
	Pottiaceae	*Tortella tortuosa* (Hedw.) Limpr.	Lima 806 (SP)
	Priodontaceae	*Prionodon densus* (Hedw.) Müll.Hal.	Yano 13579 (SP)
	Pterobryaceae	*Orthostichopsis tortipilis* (Müll.Hal.) Broth.	Peralta 27515 (SP)
	Ptychomitriaceae	*Ptychomitrium sellowianum* (Müll.Hal.) A.Jaeger	Yano 12555 (SP)
	Pylaisiadelphaceae	*Isopterygium tenerifolium* Mitt.	Peralta 20516 (SP)
	Pylaisiadelphaceae	*Isopterygium tenerum* (Sw.) Mitt.	Eiten 7100
	Racopilaceae	*Racopilum tomentosum* (Hedw.) Brid.	Alkimim-Faria 1402 (UB)
	Rhacocarpaceae	*Rhacocarpus inermis* (Müll.Hal.) Lindb.	Vital 1192 (SP)
	Rhacocarpaceae	*Rhacocarpus purpurascens* (Brid.) Müll.Hal.	Peralta 27643 (SP)
	Rhizogoniaceae	*Pyrrhobryum spiniforme* (Hedw.) Mitt.	Gonçalves 314 (SP)
	Rhizogoniaceae	*Rhizogonium novae-hollandiae* (Brid.) Brid.	Schaefer-Verwimp 13122 (SP)
	Rigodiaceae	*Rigodium toxarion* (Schwägr.) A.Jaeger.	Peralta 27537 (SP)
	Seligeriaceae	*Brachydontium notorogenes* W.R.Buck & Schaf.-Verw.	Peralta 20441 (SP)
	Sematophyllaceae	*Aptychella proligera* (Broth.) Herzog	Peralta 27650 (SP)
	Sematophyllaceae	*Aptychopsis pungifolia* (Hampe) Broth.	Gonçalves 200 (SP)
	Sematophyllaceae	*Aptychopsis pyrrhophylla* (Müll.Hal.) Wijk & Margad.	Peralta 27485 (SP)
	Sematophyllaceae	*Brittonodoxa subpinnata* (Brid.) W.R.Buck, P.E.A.S.Câmara & Carv.-Silva *	Müller (1845)
	Sematophyllaceae	*Donnellia commutata* (Müll.Hal.) W.R.Buck	Alkimim-Faria 1342 (UB)
	Sematophyllaceae	*Donnellia lagenifera* (Müll.Hal.) W.R.Buck	Gonzatti 4005 (SP)
	Sematophyllaceae	*Microcalpe subsimplex* (Hedw.) W.R.Buck *	Müller (1845)
	Sematophyllaceae	*Kuerschneria laevigata* (Herzog) Ochyra & Bedn.-Ochyra *	Ochyra & Bednarek Ochyra (2010) [53]
	Sematophyllaceae	*Sematophyllum beyrichii* (Hornsch.) Broth.	Gonçalves 331 (SP)
	Sematophyllaceae	*Sematophyllum cuspidiferum* Mitt.	Peralta 20495 (SP)
	Sematophyllaceae	*Sematophyllum decumbens* Mitt.	Peralta 27494 (SP)
	Sematophyllaceae	*Sematophyllum galipense* (Müll.Hal.) Mitt.	Peralta 27748 (SP)
	Sematophyllaceae	*Sematophyllum lithophilum* (Hornsch.) Ångström *	Buck (1998) [54]
	Sematophyllaceae	*Sematophyllum subdepressum* (A.Jaeger) Broth. *	Brotherus (1924)
	Sematophyllaceae	*Sematophyllum subpinnatum* (Brid.) E.Britton	Peralta 27743 (SP)
	Sematophyllaceae	*Trichosteleum glaziovii* (Hampe) W.R.Buck	Peralta 27514 (SP)
	Sematophyllaceae	*Trichosteleum sentosum* (Sull.) A.Jaeger	Peralta 27497 p.p. (SP)
	Sematophyllaceae	*Vitalia cuspidifera* (Mitt.) P.E.A.S.Câmara, Carv.-Silva & W.R.Buck	Sampaio, A.J. 2380
	Sematophyllaceae	*Vitalia galipensis* (Müll.Hal.) P.E.A.S.Câmara, Carv.-Silva & W.R.Buck	Lima 768 p.p. (SP)
	Sphagnaceae	*Sphagnum aciphyllum* Müll.Hal	Peralta 27592 (SP)
	Sphagnaceae	*Sphagnum brasiliense* Warnst.	Santos & Costa 759, 760 (RB)
	Sphagnaceae	*Sphagnum brevirameum* Hampe	Gonçalves 167 (SP)
	Sphagnaceae	*Sphagnum capillifolium* (Ehrh.) Hedw.	Gonçalves 170 (SP)
	Sphagnaceae	*Sphagnum costae* H.A.Crum & D.P.Costa	Peralta 20494 (SP)
	Sphagnaceae	*Sphagnum cuspidatum* Ehrh. ex Hoffm. *	Luetzelburg (1923, as *S. serratum*) Gonçalves 369 (SP)
	Sphagnaceae	*Sphagnum divisum* H.A.Crum	Gonçalves 419 (SP)
	Sphagnaceae	*Sphagnum exquisitum* H.A.Crum	Lima 822 (SP)
	Sphagnaceae	*Sphagnum geraisense* H.A.Crum	Gonçalves 421 (SP)
	Sphagnaceae	*Sphagnum gracilescens* Hampe ex. Müll.Hal.	Peralta 27651 (SP)
	Sphagnaceae	*Sphagnum luetzelburgii* H.K.G.Paul ex H.A.Crum	Costa 4173 (RB)
	Sphagnaceae	*Sphagnum longicomosum* Müll.Hal. ex Warnst.	Gonçalves 335 (SP)
	Sphagnaceae	*Sphagnum longistolo* Müll.Hal.	Peralta 27553 (SP)
	Sphagnaceae	*Sphagnum magellanicum* Brid.	Lima 824 (SP)
	Sphagnaceae	*Sphagnum palustre* L. *	Andrews (1941) [55]
	Sphagnaceae	*Sphagnum perforatum* Warnst.	Costa et al. 5017 (RB)
	Sphagnaceae	*Sphagnum perichaetiale* Hampe	Peralta 27652 (SP)
	Sphagnaceae	*Sphagnum platyphylloides* Warnst.	Gonçalves 415 (SP)
	Sphagnaceae	*Sphagnum pseudoramulinum* H.A.Crum	Lima 817 (SP)
	Sphagnaceae	*Sphagnum ramulinum* Warnst.	Lima 815 (SP)
	Sphagnaceae	*Sphagnum recurvum* P.Beauv.	Peralta 27560 (SP)
	Sphagnaceae	*Sphagnum sanguinale* Warnst.	Gonçalves 168 (SP)
	Sphagnaceae	*Sphagnum sehnemii* H.A.Crum	Luetzelburg 6162 (F, HBR as *Sphagnum densum*)
	Sphagnaceae	*Sphagnum subsecundum* Nees	Gonçalves 353 (SP)
	Sphagnaceae	*Sphagnum sucrei* H.A.Crum	Peralta 27598 (SP)
	Stereophyllaceae	*Entodontopsis leucostega* (Brid.) W.R.Buck & Ireland	Alkimim-Faria 1456 (UB)
	Stereophyllaceae	*Entodontopsis nitens* (Mitt.) W.R.Buck & Ireland	Alkimim-Faria 1263 (UB)
	Stereophyllaceae	*Eulacophyllum cultelliforme* (Sull.) W.R.Buck & Ireland *	Costa et al. (2005) [37]
	Thuidiaceae	*Thuidium pseudoprotensum* (Müll.Hal.) Mitt.	Peralta 27616 (SP)
	Thuidiaceae	*Thuidium recognitum* (Hedwig) Lindberg	Lima 797 (SP)
	Thuidiaceae	*Thuidium tamariscinum* (Hedw.) Schimp.	Gonçalves 359 (SP)
	Thuidiaceae	*Thuidium tomentosum* Schimp.	Peralta 20510 (SP)
	Thuidiaceae	*Pelekium involvens* (Hedw.) A. Touw	Lima 751 (SP)
	Thuidiaceae	*Pelekium minutulum* (Hedw.) A.Touw	Lima 746 (SP)
	Thuidiaceae	*Pelekium muricatulum* (Hampe) A.Touw	Alkimim-Faria 1376 (UB)
	Thuidiaceae	*Pelekium scabrosulum* (Mitt.) A.Touw	Alkimim-Faria 1388 (UB)
**Hornworts**	Anthocerotaceae	*Anthoceros hispidus* Steph.	Yano 13588 (SP)
	Anthocerotaceae	*Anthoceros punctatus* L. *	Hooker & Wilson (1844) [56]
	Dendrocerotaceae	*Dendroceros crispatus* (Hook.) Nees	Peralta 27459 (SP)
	Dendrocerotaceae	*Dendroceros crispus* (Sw.) Nees *	Gottsche et al. (1844)
	Dendrocerotaceae	*Nothoceros minarum* (Nees) J.C.Villarreal	Gonçalves 178 SP)
	Dendrocerotaceae	*Nothoceros vincentianus* (Lehm. & Lindenb.) J.C. Villareal	Peralta 27705 (SP)
	Notothyladaceae	*Phaeoceros bulbiculosus* (Broth.) Prosk.	Peralta 20452 (SP)
	Nothotyladaceae	*Phaeoceros laevis* (L.) Prosk.	Peralta 27542 (SP)

**Table 2:** List of endemic bryophyte species found in PARNASO. (EB = endemic to Brazil; ERJ = endemic to Rio de Janeiro State; and CS = conservation status category.

Group	Táxon	EB	ERJ	CS
**Liverworts**	*Cheilolejeunea grosseoleosa* C.J.Bastos & Schäf.-Verw.	X		
	*Cololejeunea diaphana* A.Evans	X		
	*Cololejeunea papilliloba* (Steph). Steph.	X		
	*Colura itatyana* Steph.	X	X	
	*Frullania schaefer-verwimpii* Yuzawa & Hatt.	X		
	*Frullania vitalii* Yuzawa & Hatt.	X		
	*Fuscocephaloziopsis crassifolia* (Lindenb. & Gottsche) Váňa & L. Söderstr.	X		
	*Gongylanthus liebmannianus* (Lindenb. & Gottsche) Steph.			EN
	*Haplolejeunea umbrosa* Gradst. & Ilkiu-Borges	X		
	*Harpalejeunea schiffneri* S.W. Arnell	X		
	*Harpalejeunea subacuta* A.Evans	X		
	*Lejeunea beyrichiana* (Steph) Gradst. & C.J.Bastos	X	X	
	*Lejeunea longidentata* C.J.Bastos, Gradst., S. Vilas Bôas-Bastos & Schäf.-Verw.	X		
	*Lejeunea oligoclada* Spruce	X		
	*Leptoscyphus spectabilis* (Steph.) Grolle	X		
	*Metzgeria brasiliensis* Schiffn.	X		
	*Metzgeria convoluta* Steph.	X		
	*Metzgeria cratoneura* Schiffn.	X		
	*Metzgeria hegewaldii* Kuwah.		X	EN
	*Microlejeunea squarrosa* J. Heinrichs, A. Schäfer-Verwimp, Pócs & S.S. Dong	X		
	*Micropterygium pterygophyllum* (Nees)	X		
	*Neesioscyphus carneus* (Nees) Grolle	X		
	*Paracromastigum dusenii* (Steph.) R.M.Schust.		X	EN
	*Plagiochila amicta* Steph.	X		
	*Plagiochila boryana* Gottsche ex Steph.			EN
	*Saccogynidium caldense* (Ångstr.) Grolle	X		
	*Southbya organensis* Herzog		X	CR
	*Syzygiella uleana* Steph.	X		
	*Vitalianthus bischlerianus* (Porto & Grolle) R.M.Schust. & Giancotti	X		
Mosses	*Atractylocarpus brasiliensis* (Müll.Hal.) R.S.Williams	X		EN
	*Atractylocarpus longisetus* (Hook.) E.B.Bartram			EN
	*Brachydontium notorogenes* W.R.Buck & Schaf.-Verw.	X	X	CR
	*Brachymenium hornschuchianum* Mart.	X		
	*Breutelia grandis* (Hampe) Paris	X		
	*Callicostella martiana* (Hornsch.) A.Jaeger	X		
	*Calyptrochaeta albescens* (Hampe) W.R.Buck	X		
	*Campylopus densicoma* (Müll.Hal.) Paris			EN
	*Campylopus dichrostis* (Müll.Hal.) Paris	X		
	*Campylopus gemmatus* (Müll.Hal.) Paris	X		
	*Campylopus julicaulis* Broth.	X		
	*Campylopus thwaitesii* (Mitt.) A.Jaeger	X		
	*Campylopus uleanus* (Müll.Hal.) Broth.	X		
	*Campylopus widgrenii* (Müll.Hal.) Mitt.	X		
	*Cladastomum robustum* Broth.	X		
	*Cladastomum ulei* Müll.Hal.	X		
	*Crumuscus vitalis* W.R.Buck	X		
	*Cyclodictyon marginatum* (Hook. & Wilson) Kuntze	X		
	*Daltonia splachnoides* (Sm.) Hook. & Taylor	X		
	*Dicranodontium pulchroalare* Broth.	X	X	
	*Dimerodontium pellucidum* Schwägr.	X		
	*Ditrichum paulense* Geh. ex Hampe	X		
	*Itatiella ulei* (Broth. ex Müll.Hal.) G.L.Sm.	X		
	*Leiomela piligera* (Hampe) Broth.	X		
	*Lepidopilidium brevisetum* (Hampe) Broth.	X		
	*Lepidopilum scabrisetum* (Schwägr.) Steere	X		
	*Leptodontium stellatifolium* (Hampe) Broth.	X		
	*Leptodontium wallisii* (Müll.Hal.) Kindb.			VU
	*Leskeodon aristatus* (Geh. & Hampe) Broth.	X		
	*Leucobryum clavatum* Hampe	X		
	*Leucoloma triforme* (Mitt.) A.Jaeger	X		
	*Microcalpe subsimplex* (Hedw.) W.R.Buck	X		
	*Polytrichum angustifolium* Mitt.	X		
	*Rhacocarpus inermis* (Müll.Hal.) Lindb.	X		
	*Sphagnum brasiliense* Warnst.	X		
	*Sphagnum costae* H.A.Crum & D.P.Costa	X		
	*Sphagnum divisum* H.A.Crum	X		
	*Sphagnum exquisitum* H.A.Crum	X		
	*Sphagnum geraisense* H.A.Crum	X		
	*Sphagnum gracilescens* Hampe	X		
	*Sphagnum longicomosum* Müll.Hal. ex Warnst.	X		
	*Sphagnum luetzelburgii* H.K.G.Paul ex H.A.Crum	X	X	
	*Sphagnum perforatum* Warnst.	X		VU
	*Sphagnum pseudoramulinum* H.A.Crum	X		
	*Sphagnum ramulinum* Warnst.	X		
	*Sphagnum sehnemii* H.A.Crum	X		
	*Sphagnum sucrei* H.A.Crum	X		
	*Trichosteleum glaziovii* (Hampe) W.R.Buck	X		

## Data Availability

The original contributions presented in this study are included in the article; further inquiries can be directed to the corresponding author.

## Abbreviation

PARNASO	Serra dos Órgãos National Park
